# Supplementary material for: Fast and scalable Wasserstein-1 neural optimal transport solver for single-cell perturbation prediction
Source: Bioinformatics. 2025 Jul 15;41(Suppl 1):i513–22. doi: 10.1093/bioinformatics/btaf253 (PMC12261427; doi:10.1093/bioinformatics/btaf253)
Supplement: btaf253_Supplementary_Data [file btaf253_supplementary_data.pdf]

Supplementary file for Fast and scalable Wasserstein-1 neural optimal transport solver for single-cell perturbation prediction

Yanshuo Chen[1,2], Zhengmian Hu[1], Wei Chen[3,4], and Heng Huang[1,2]
[1] Department of Computer Science, University of Maryland, College Park, MD, 20742, USA.
[2] Center of Bioinformatics and Computational Biology, College Park, MD, 20740, USA.
[3] Department of Biostatistics, University of Pittsburgh, Pittsburgh, 15261, PA, USA
[4] Department of Pediatrics, UPMC Children's Hospital of Pittsburgh, Pittsburgh, 15224, PA, USA

Table of contents

Supplementary Figures..2

Supplementary Figures

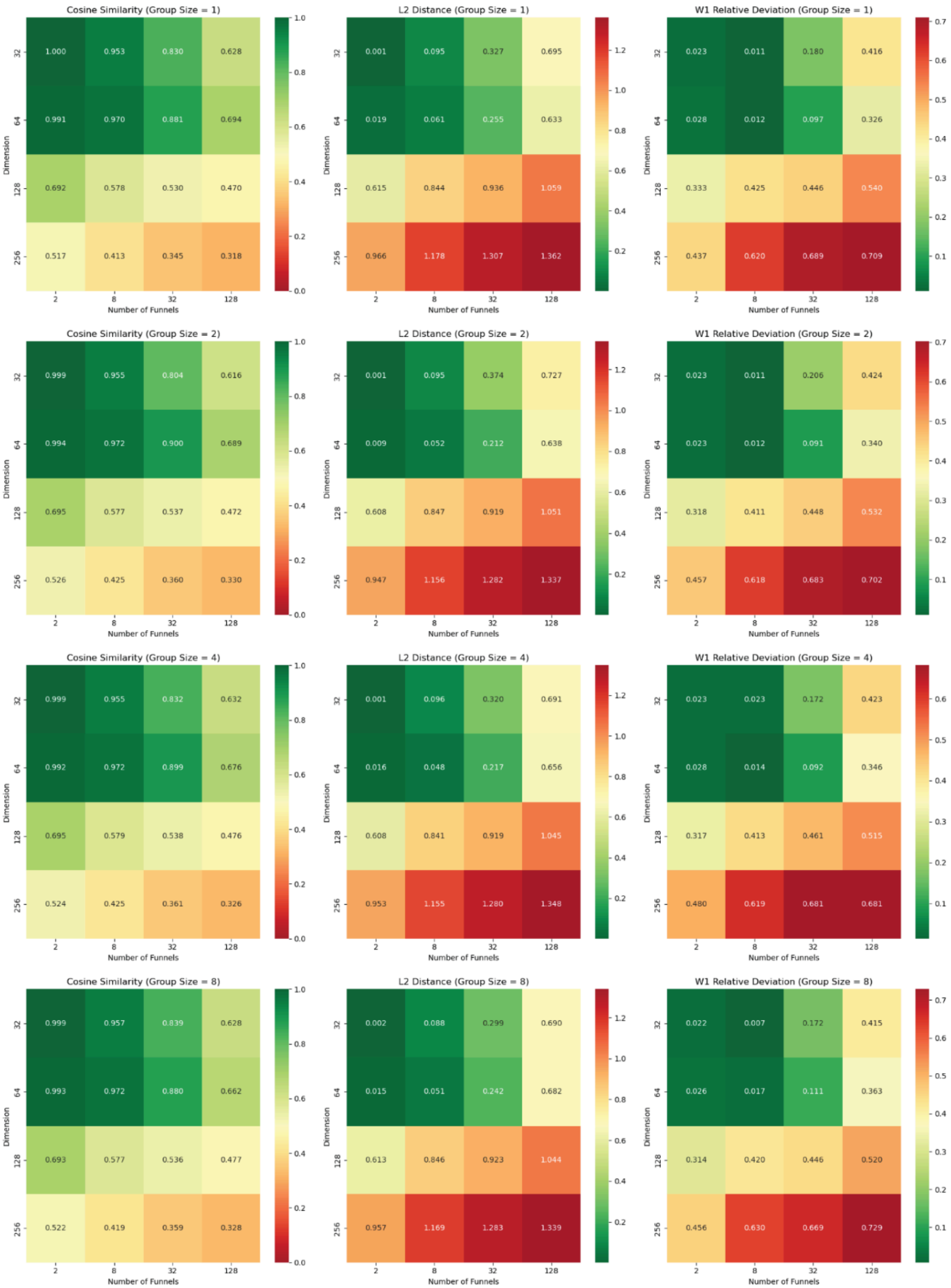
Cosine Similarity (Group Size = 1)
1.000 0.953 0.830 0.628
0.991 0.970 0.881 0.694
0.692 0.578 0.530 0.470
0.517 0.413 0.345 0.318
L2 Distance (Group Size = 1)
0.001 0.095 0.327 0.695
0.019 0.061 0.255 0.633
0.615 0.844 0.936 1.059
0.966 1.178 1.307 1.362
W1 Relative Deviation (Group Size = 1)
0.023 0.011 0.180 0.416
0.028 0.012 0.097 0.326
0.333 0.425 0.446 0.540
0.437 0.620 0.689 0.709
Cosine Similarity (Group Size = 2)
0.999 0.955 0.804 0.616
0.994 0.972 0.900 0.689
0.695 0.577 0.537 0.472
0.526 0.425 0.360 0.330
L2 Distance (Group Size = 2)
0.001 0.095 0.374 0.727
0.009 0.052 0.212 0.638
0.608 0.847 0.919 1.051
0.947 1.156 1.282 1.337
W1 Relative Deviation (Group Size = 2)
0.023 0.011 0.206 0.424
0.023 0.012 0.091 0.340
0.318 0.411 0.448 0.532
0.457 0.618 0.683 0.702
Cosine Similarity (Group Size = 4)
0.999 0.955 0.832 0.632
0.992 0.972 0.899 0.676
0.695 0.579 0.538 0.476
0.524 0.425 0.361 0.326
L2 Distance (Group Size = 4)
0.001 0.096 0.320 0.691
0.016 0.048 0.217 0.656
0.608 0.841 0.919 1.045
0.953 1.155 1.280 1.348
W1 Relative Deviation (Group Size = 4)
0.023 0.023 0.172 0.423
0.028 0.014 0.092 0.346
0.317 0.413 0.461 0.515
0.480 0.619 0.681 0.681
Cosine Similarity (Group Size = 8)
0.999 0.957 0.839 0.628
0.993 0.972 0.880 0.662
0.693 0.577 0.536 0.477
0.522 0.419 0.359 0.328
L2 Distance (Group Size = 8)
0.002 0.088 0.299 0.690
0.015 0.051 0.242 0.682
0.613 0.846 0.923 1.044
0.957 1.169 1.283 1.339
W1 Relative Deviation (Group Size = 8)
0.022 0.007 0.172 0.415
0.026 0.017 0.111 0.363
0.314 0.420 0.446 0.520
0.456 0.630 0.669 0.729
Dimension
32
64
128
256
Number of Funnels
2
8
32
128

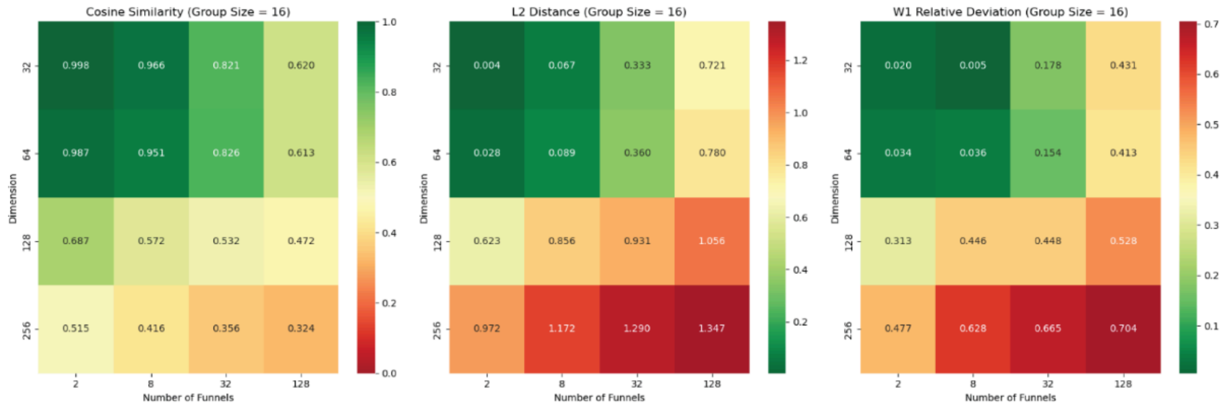

Fig. S1. Group sort network's performance with different group size. The performance of a Wasserstein-1 solver can be assessed by the cosine similarity and distance between the estimated direction and the true direction. It can also be tested by showing the deviation between estimated W1 distance and the true W1 distance. Number of funnels represents the number of modes in the synthetic data, more funnels makes the task more difficult. Dimension refers to the data feature dimension. This benchmark shows that group size = 4 has the best estimated W1 distance in high dimensional high complexity setting. Hence, we use the group size = 4 throughout the paper.

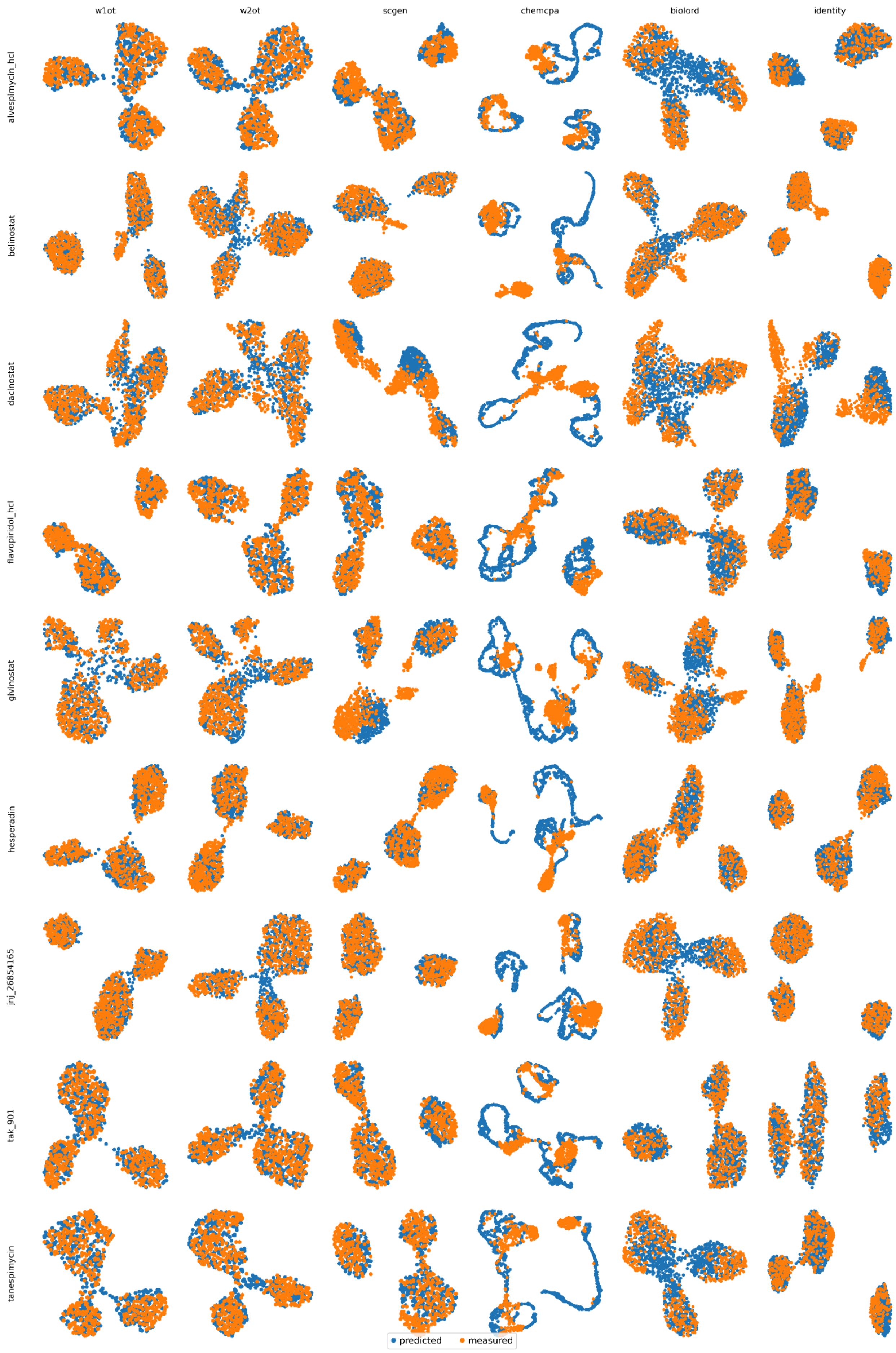

w1ot
w2ot
scgen
chemcpa
biolord
identity
alvespimycin_hcl
belinostat
dacinostat
flavopiridol_hcl
givinostat
hesperadin
jnj_26854165
tak_901
tanespimycin
predicted
measured

Fig. S2. UMAP visualization of each method's prediction and the ground truth on sciplex3 dataset. The results indicate that the OT methods surpasses other methods in terms of distribution alignment.

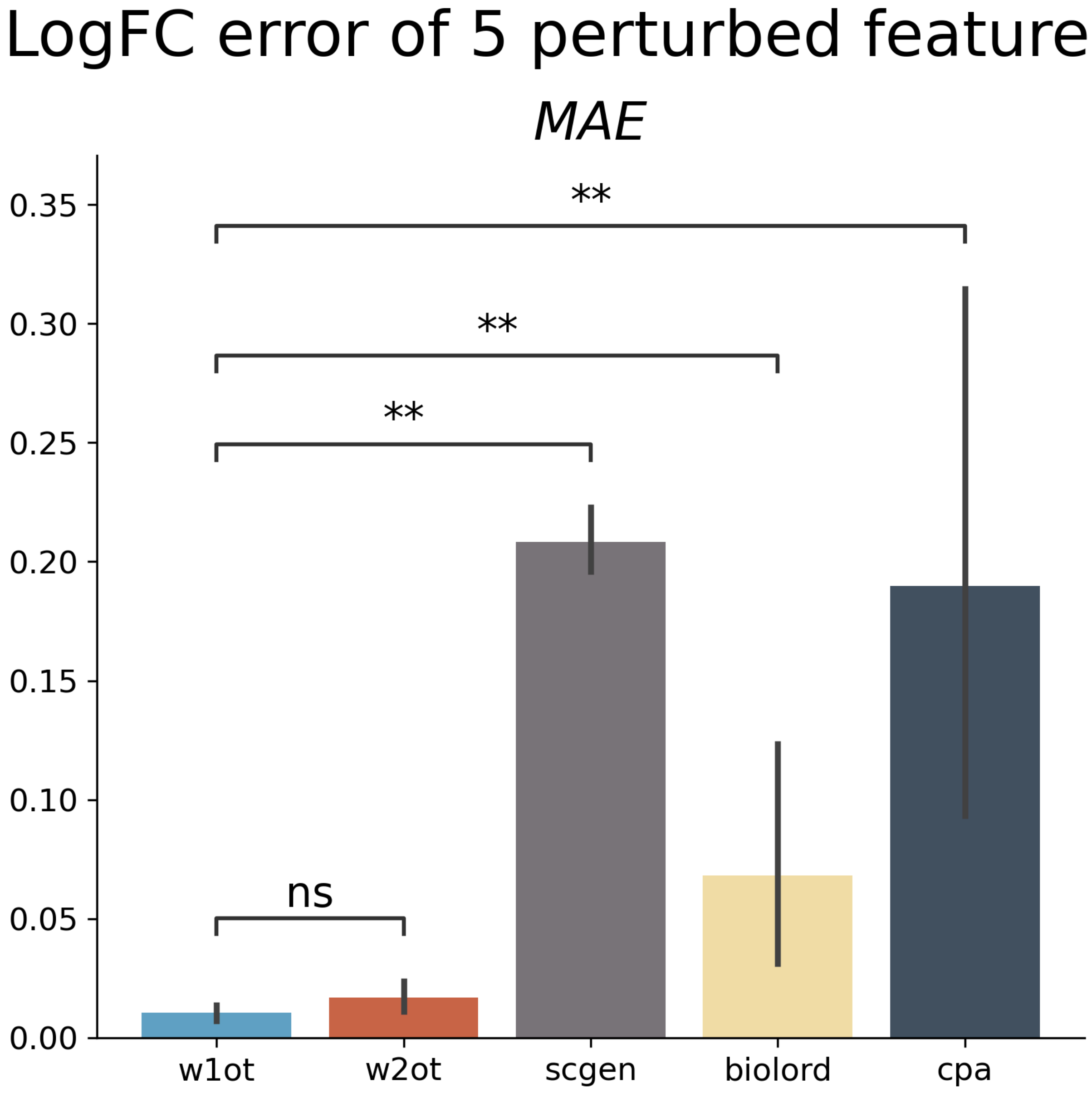

Fig. S3. Mean absolute log fold change (logFC) error for 5 perturbed features across different models. We perturbed 5 randomly selected genes in the 4i dataset and evaluated model performance by measuring the mean absolute error (MAE) between predicted and actual logFCs. Lower values indicate better accuracy in capturing perturbation effects. Statistical significance is indicated by asterisks ($p < 0.01$; ns = not significant).
